# Ischemic stroke and repair: current trends in research and tissue engineering treatments

**DOI:** 10.1186/2050-490X-2-3

**Published:** 2014-02-03

**Authors:** Jian Wang, Wen Yang, Hongjian Xie, Yu Song, Yongkui Li, Lin Wang

**Affiliations:** Center for Tissue Engineering and Regenerative Medicine, Union Hospital, Tongji Medical College, Huazhong University of Science and Technology, Wuhan, China; Medical Research Center, Union Hospital, Tongji Medical College, Huazhong University of Science and Technology, Wuhan, China

**Keywords:** Ischemic stroke, Tissue engineering, Biomaterials, Neuro-protective factors, Cell therapy

## Abstract

Stroke, the third leading cause of mortality, is usually associated with severe disabilities, high recurrence rate and other poor outcomes. Currently, there are no long-term effective treatments for stroke. Cell and cytokine therapies have been explored previously. However, the therapeutic outcomes are often limited by poor survival of transplanted cells, uncontrolled cell differentiation, ineffective engraftment with host tissues and non-sustained delivery of growth factors. A tissue-engineering approach provides an alternative for treating ischemic stroke. The key design considerations for the tissue engineering approach include: choice of scaffold materials, choice of cells and cytokines and delivery methods. Here, we review current cell and biomaterial based therapies available for ischemic stroke, with a special focus on tissue-engineering strategies for regeneration of stroke-affected neuronal tissue.

## Review

### Introduction

Stroke is the third leading cause of disease mortality worldwide. In the United States, about 150,000 individuals die of stroke every year [1]. Ischemic stroke is caused by interruption of the cerebral blood supply, accounting for about 80% of all stroke cases [2]. Depending on the regions where ischemic stroke occurs, it can impair patients’ capabilities of sensory processing, communication, cognition and motor function [3]. Motor impairment associated with stroke often leads to short-term or permanent disabilities, substantially affecting patients’ life quality [4]. In addition, stroke increases the risk of Parkinson’s disease and Alzheimer’s disease [5].

Currently, there are no long-term effective clinical treatments available for stroke as few of them leads to complete functional recovery [6, 7]. The intravenous administration of tissue-type plasminogen activator (tPA) is a proven intervention for acute ischemic stroke patients [8]. However, this treatment is only applicable to a small percentage of stroke patients in the acute phase as its therapeutic time window is rather narrow (up to 4.5 hours after the onset of symptoms) [9]. Meanwhile, this treatment was reported to increase the risk of intracranial hemorrhage [10]. Physical therapy is often used to restore motor function after stroke [4, 11, 12]. However, 15–30% of stroke patients are still permanently disabled even with intensive task-­specific training [4, 13]. Early motor training and physical therapy might impede functional recovery and enlarge lesion size as suggested by animal experiments [14, 15]. Thus, few of these treatments can lead to complete functional recovery. Cell and cytokine therapies [16–23] have been explored previously. The regeneration outcome of this therapy is often limited due to poor cell survival, uncontrolled *in vivo* differentiation of delivered cells, ineffective integration of delivered cells with the host tissue and non-sustained delivery of growth factors.

These limitations may be overcome by employing a tissue engineering strategy. Tissue-engineered scaffolds can provide a highly biocompatible three-dimensional environment that supports the long-term growth of therapeutic cells seeded on the scaffolds, thus improving the *in vivo* survival of these cells. Meanwhile, the scaffold can act as a drug delivering vehicle releasing neuro-protective factors in a controlled manner, which promotes the regeneration and functional recovery of damaged neuronal tissue [16]. In this review, we summarize current therapies for ischemic stroke, with a special emphasis on the important aspects of the tissue-engineering strategy for stroke treatment.

### Cell therapy

Various types of cells have been investigated for their potential to regenerate damaged neural tissue caused by ischemic stroke (Table 1).

**Table 1:** **Cell therapy used in the treatment of stroke**

Stem cell	Source	Function	Species	Model	Effects	Refs.
Neural stem cells	Neuraxis of adult CNS	Differentiate into three CNS cell types in stroke-damaged brain, including neurons, astrocytes, and oligodendrocytes	Rat	MCAO	Behavioral recovery on a series of sensory tasks and motor tasks; neural stem cells differentiated into neurons and promote function recovery	[24–30]
Mesenchymal stem cells	Bone marrow	Differentiate into osteoblasts, chondroblasts, adipocytes, neurons and some other cell types	Rat	MCAO	Behavioral recovery; facilitated functional recovery; reduced scar thickness and increased number of oligodendrocyte precursor cells and proliferating cells along SVZ	[31–34]
Olfactory Ensheathing cells	Nasal olfactory mucosa	Guide axon outgrowth and remyelinate axons and secrete many trophic factors (including BDNF, VEGF and glial cell line-derived neurotrophic factor (GDNF)	Rat	MCAO	The combined transplantation of OECs with fibroblasts facilitated neurite outgrowth and led to a reversal of the neurological deficits	[35–39]
Dental stem cells	Dental pulp (dental pulp stem cells) as well as dental follicle cells	Differentiate into neural cells, osteocytes, adipocytes, chondrocytes, muscle cells and hepatocytes *in vitro* and *in vivo*; express neurotrophic factors such as GDNF, BDNF and nerve growth factor (NGF); promote angiogenesis	Rat	MCAO	Functional recovery was observed in one motor task; surviving cells may have differentiated into neurons	[40–43]

### Neural stem cells

Neural stem cells (NSCs) can be obtained from along the entire neuraxis of adult central nervous system (CNS) [24–27]. NSCs have been experimentally utilized to treat CNS disorders, including stroke [28, 44]. The intracranial injection of NSCs isolated from rat subventricular zone (SVZ) into a rat stroke model, middle cerebral artery occlusion (MCAO), led to sensory and motor recovery [28].

Human neural stem cells (hNSCs) have also been applied into the treatment of neuronal disorders. These cells are isolated from the embryonic or fetal CNS [44]. The human neurospheres derived from these hNSCs can survive robustly in naive and ischemic brains [20]. When hNSCs were injected directly into the stroke-damaged striatum of adult rats, these cells differentiated and expressed mature neuronal markers, such as calbindin, HuD and parvalbumin [45], indicating that transplanted NSCs remain competent to differentiate into functional neurons. This result is consistent with another study, which showed that after implanted into the damaged central nervous system of ischemic rats, a fraction of the grafted neural stem cells were able to differentiate into neurons *in vivo* and promote function recovery [29]. Although NSCs showed a great potential in treating cerebral ischemic lesion, many problems remain, for instance, the low *in vivo* survival rate of these cells, immune rejection and ethical issues.

### Mesenchymal stem cells

Mesenchymal stem cells (MSCs), presented in the bone marrow, are multipotent adult stem cells with the capabilities of differentiating into various cell types, including neurons [31, 32]. MSCs can differentiate into neurons *in vitro* in an experimentally controlled manner. When cultured with differentiation factors (e.g. β-mercaptoethanol and dimethylsulfoxide) [32], growth factors (e.g. fibroblast growth factor-2 (FGF2) and epidermal growth factor (EGF) [46], brain-derived neurotrophic factor (BDNF) [47] or retinoic acid (RA) [48]), these MSCs express neuronal markers (neuron-specific endonuclease, NeuN neurofilament-M, glial fibrillary acidic protein (GFAP), tyrosine hydroxylase, and β-III-tubulin) [46]. MSCs were reportedly to be able to survive and migrate into lesion sites after transplanted into the experimental models of stroke [49]. Moreover, the intravenous injection of MSCs was found to promote functional recovery in the animal stroke MCAO model [33]. This function recovery resulted from MSCs treatment may be associated with reduced scar thickness and the increased number of oligodendrocyte precursor cells and proliferating cells along the SVZ [34]. Notably, the therapeutic effects of MSCs on stroke have been recently evaluated in several clinical trials. In a small trial, the intravenous infusion of autologous MSCs significantly improved functional recovery without obvious adverse effects during one-year follow-up [50]. Similar recovery effects were also observed in a long-term clinical trial with 52 stroke patients [51]. Compared with NSCs, MSCs appear to be more conveniently obtained. Autologous MSCs can reduce immunologic rejection and bypass ethical issues. Nonetheless, like NSCs, the application of MSCs for stroke treatment faces the similar challenges, such as poor *in vivo* cell viability.

### Stimulation of endogenous neurogenesis

While stroke patients may benefit from neuronal regeneration mediated by exogenously delivered stem cells, stimulating endogenous neurogenesis by activating brain resident cells for neuronal repair can be another potential approach for treating stroke. In response to stroke or other brain injury, the degree of endogenous neurogenesis, neurite outgrowth and functional recovery are often constrained [52–56]. There are two main regions of the adult brain that contain proliferating progenitor cells: the SVZ and the subgranular zone (SGZ) [57]. While under normal conditions the quiescent ependymal cells do not contribute to neurogenesis, these cells can be activated to give rise to neuroblasts and astrocytes in response to stroke [58]. One way to stimulate endogenous neurogenesis in the SVZ is reportedly to use BDNF fused with a collagen-binding domain (CBD-BDNF) as a stimulant [59]. The injection of CBD-BDNF into the lateral ventricle of MCAO rats was shown to promote local neural regeneration, angiogenesis and improve functional recovery [59].

### Induced pluripotent stem cells

Dating back to the recent breakthroughs in the stem cell field, induced pluripotent stem (iPS) cells, one of the most exciting improvements, have been generated from a host of human somatic cells since their original characterization in 2007 [60]. The obvious similarities they share with embryonic stem cells, including the ability to differentiate into all cell types composing the tissues of the body, turn out to be much remarkable.

It seems more attractive to clinical applications as iPS cells generated from a patient can be applied for treatment of various diseases in an autologous manner. Transplantation of iPS cells has been experimentally tested for treating stroke. A mixture containing iPS cells and fibrin glue was delivered into the subdural space following MCAO. This treatment decreased total infarct volume and significantly improved the motor function (rotarod and grasping tasks) [61]. A notable reduction in pro-inflammatory cytokines and a simultaneous increase in the anti-inflammatory cytokines were also observed [61]. However, another study showed the formation of tridermal teratoma in the brain after being transplanted with undifferentiated iPS cells into the ipsilateral striatum and the cortex of rats following the transient MCAO [62]. Although the control over *in vivo* differentiation of iPS cells needs further study, it is undeniable that iPS cells will continue to be a focus of the cell therapy for a variety of neurodegenerative diseases, including stroke [63].

## Biomaterials

A plethora of biomaterials have been investigated for their potential to treat stroke (Table 2) [64]. An ideal biomaterial for stroke treatment is expected to meet several requirements: first, it must have specific physical and biochemical properties allowing cell to attach to it, proliferate and differentiate on it, migrate off to integrate with host neuronal tissue [16, 17]. Second, it should degrade *in vivo* to permit tissue healing and growth. Moreover, its degradation products should be highly biocompatible, non-cytotoxic, non-inflammatory and non-hemolytic [16], causing no adverse effects on implanted tissues. This biomaterial can be designed to encapsulate drugs or molecules. The advantages of the encapsulation of therapeutic chemicals are obvious, including stabilizing the drugs that may have a short half-life, enabling the controlled release of therapeutic factors, limiting additional damages to healthy tissues and reducing side effects [65–67]. A number of biomaterials have been explored for the repair of ischemic lesion in animal models (Table 2). Here, we will explore these biomaterial-based therapies for stroke with the emphasis on the materials.

**Table 2:** **Biomaterials used in the treatment of stroke**

Materials	Species	Model	Effects	Functional recovery	Refs.
HAMC + PLGA + EGF-PEG + EPO	Mouse	Endothelin-1 induced small cortical infarcts	Led to neural tissue repair	N.A.	[68]
PLGA-PEG + T_3_	Mouse	MCAO	A 34% decrease in tissue infarction and a 59% decrease in brain edema	N.A.	[69]
Collagen type I + NSCs	Rat	MCAO	NSCs survived, differentiated and formed synapses in the brain	Function outcome is improved in neurological severity score	[70]
Hyaluronan-Heparin-Collagen + neural progenitor cells (NPCs)	Mouse	Photochemically induced cerebral ischemic	Promoted survival of NPCs and diminished the infiltration of Microglia/Macrophage cells	N.A.	[71]
Alginate + VEGF	Rat	MCAO	Reduced the lesion volume	Function outcome is improved in bias swing test and neurological severity score	[72]
PLGA + hNSCs + VEGF	Rat	MCAO	Attracted host endothelial cells (ECs) and developed a vascular network within *de novo* tissue	N.A.	[22]
HAMC + EPO	Mouse	Endothelin-1 induced small cortical infarc	Attenuated inflammatory responses; reduced stroke cavity size; increased the number of neurons and decreased apoptosis	N.A.	[73]
Hyaluronic-Acid(HA)-based hydrogel + Nogo-66 receptor (NgR)	Rat	MCAO	Supported cell migration, development and neural regeneration in the brain	Ameliorated the disabled function of the impaired forelimb	[74]
PGA + NSCs	Mouse	Hypoxia induced ischemic	Promoted neuronal differentiation; enhanced elaboration of neural processes; fostered re-formation of cortical tissue and reduced inflammation and scarring	N.A.	[75]

### HAMC

Owing to versatile forms and composition, polymer-based hydrogels have been widely used in many fields, including neural tissue engineering [16, 76–78]. Hydrogels are polymeric materials with high water content (i.e. >90% water) and diverse physical properties [79]. Hyaluronan/methyl cellulose (HAMC), an injectable hydrogel, has been used to achieve a short-term controlled delivery of erythropoietin (EPO) in the stroke treatment [73]. The local release of EPO from this HAMC was found to promote endogenous neurogenesis of the SVZ and tissue repair after stroke injury in the mouse brain.

### Alginate

Alginate is naturally derived polysaccharides from brown algae [80] and has been extensively used as hydrogel synthetic extracellular matrix (ECM) [81–84]. As a biomaterial, alginate is applied in pharmaceutical industry due to its mechanical and chemical stability and high biocompatibility [85].The utilization of an alginate hydrogel encapsulating VEGF was shown to induce structural and functional protection from ischemic stroke damage in the rat MCAO model [72].

### Collagen

Collagen, the main component of the extracellular matrix, can provide a proper surface for cell adhesion and migration [86]. Because of its advantages in biological compatibility, mechanical strength, degradability and immunogenicity [87, 88], collagen has been widely used in biomedical applications, including stroke treatment. A hyaluronan-heparin-collagen hydrogel seeded with stem cells was transplanted into the infarct cavity after stroke, leading to improved stem cell survival and reduced damage to the brain tissue [71]. When collagen type I and NSCs were combined and transplanted *in vivo* to treat cerebral ischemic injury, NSCs were found to differentiate and form new synapses. This treatment promoted the structural and functional repair of brain tissue following ischemic stroke [70].

### PLGA

Poly (D, L-lactic acid-co-glycolic acid)(PLGA) is a fully degradable biomaterial with the end products, CO_2_ and H_2_O [89]. PLGA particles can be readily obtained using a single oil-in-water emulsion technique. PLGA scafolds can act as a structural support for neural stem cells to enhance brain repair [90]. The implantation of PLGA particles into the brain does not induce adverse host cell responses, such as glial scar and inflammation [89], indicating the excellent biocompatibility of PLGA.

PLGA particles have been used as a vehicle for delivering a number of therapeutic factors for stroke treatment, for instance, PLGA-PEG encapsulating T_3_ (thyroid hormone) [69]. This PLGA-based treatment led to a decrease in tissue infarction and brain edema. In another stroke treatment, pegylated EGF (PEG-EGF) and EPO were loaded in PLGA nanoparticles and biphasic microparticles (a PLGA core and a poly(sebacic acid) shell), respectively, which were dispersed in an HAMC) hydrogel. This drug delivery system reduced the inflammatory responses and significantly improved neurogenesis [68]. In addition to carrying cytokines, PLGA particles can also be used to deliver stem cells for the therapeutic purpose. hNSCs were seeded onto VEGF-releasing PLGA particles. This cell-cytokine-biomaterial system was found to attract host endothelial cells and promote the development of a local vascular network [22].

### Neuro-protective factors

A wide range of neuro-protective factors, including BDNF, GDNF, EPO and NGF (Table 3), have been utilized in treating stroke in animal models [73, 91, 92]. These neuro-protective factors can promote neurogenesis mediated by endogenous NPCs and the survival, proliferation and differentiation of transplanted neural cells [93–96]. Thus, these factors play a critical role in stroke treatment.

**Table 3:** **Neuro**-**protective factors used in the treatment of stroke**

Species	Model	Neuro-protective factors	Effects	Functional recovery	Refs
Rat	MCAO	BDNF	Regulated neuronal survival, migration, differentiation and synaptic function; reduced infarct size	N.A.	[97]
Rat	MCAO	GDNF	Promoted neuronal survival; regulated migration and differentiation of several peripheral neurons	N.A.	[98–104]
Rat	MCAO	EPO	Promoted the differentiation and proliferation of erythroid progenitor cells; improved the survival of maturing cells; enhanced angiogenesis and neurogenesis	Improved neurological outcome on the foot fault and corner tests	[105, 106]
Rat	MCAO	EPO + hCG	Decreased the total infarct volume	Improved composite neurological score and forelimb placing behavioral outcome	[107]
Rat	PVD lesion of motor and sensory cortex	EPO + EGF	Promoted migration of SVZ NPCs to infarct sizes; differentiated into neurons and astrocytes; enhanced cortical regeneration	Showed improvement in cylinder test and swimming task	[108]
Rat	MCAO	FGF2	Reduced infarct volume; improved neurobehavioral and histological outcomes; increased the number of SVZ newborn neurons	Acquired better symmetry of movement and forepaw outstretching in aged rats	[109]
Rat	MCAO	FGF2 + platelet-poor plasma (PPP) + platelet lysate (PLT)	Increased SVZ endogenous neural stem cells (eNSC) proliferation, angiogenesis, neurogenesis and neuroprotection	Functional outcome was significantly improved for the neurological severity score	[110]
Rat	MCAO	TGF-alpha	Regulated migration and differentiation of the newly generated neurons; enhanced neurogenesis	The asymmetric behavioral outcomes were improved in the corner test and the cylinder test.	[111]
Rat	MCAO	G-CSF	Reduced infarct volume	N.A.	[112]
Rat	MCAO	NGF	Reduced apoptotic cell death after ischemic injury	N.A.	[113]

### BDNF

Brain-derived neurotrophic factor (BDNF), a secreted neurotrophin, regulates neuronal survival, migration, differentiation and synaptic function [114, 115] by binding to the tropomyosin receptor kinase B (TrkB) [116]. BDNF/TrkB signaling modulates synaptic strength and supports the survival of cortical neurons [63, 117, 118]. A number of studies have investigated the therapeutic effects of BDNF on stroke [91, 97, 119]. The different delivery methods appear to influence different aspects of the therapeutic effects of BDNF. Delivering BDNF intraventricularly reduced infarct size after focal cerebral ischemia in rats [97, 119]. In contrast, the intravenous administration of BDNF did not reduce the final infarct size, but greatly improved motor recovery and induced widespread neuronal remodeling [91]. Moreover, BDNF was also reported to protect brain tissues from ischemic injury [120].

### GDNF

Glial cell line-derived neurotrophic factor (GDNF), a member of transforming growth factor super-family named transforming growth factor-β (TGF-β) [121], is thought to be the most potent motor neurotrophic factor. It promotes neuronal survival and regulates migration and differentiation of several different types of peripheral neurons [98–104], including spinal motor neurons [122] and brain noradrenergic neurons [121]. When treated with GDNF, the infarct size and brain edema in the MCAO rats were significantly reduced [123].

### EPO

Erythropoietin (EPO), a hematopoietic cytokine [73], promotes the differentiation and proliferation of erythroid progenitor cells and improves the survival of maturing cells [105]. The treatment with recombinant human erythropoietin (rhEPO) after stroke significantly improves functional recovery and enhances angiogenesis and neurogenesis [106]. Furthermore, EPO treatment also provides neuro-protection after brain injury by decreasing the neuronal apoptosis [105]. An HAMC hydrogel encapsulating EPO decreased neuronal apoptosis and reduced stroke cavity size in the mouse brain after stroke injury [73], possibly due to attenuated inflammatory responses and enhanced neurogenesis.

### NGF

Nerve growth factor (NGF), a member of neurotrophin family, supports the growth and survival of neural cells [124]. Since it was first discovered in 1950, NGF has been explored in the regulation of developing neurological system [125]. Additionally, it also promotes the differentiation of stem cells into neurons and the migration of newly differentiated neurons [126, 127]. NGF mediates neuroprotection through proline-rich Akt substrate (PRAS) phosphorylation and its interaction with tyrosine 3-monooxygenase/tryptophan 5-monooxygenase activation protein, theta polypeptide (YWHAQ) and phosphory-lated Akt (pAkt) [113]. Several studies have investigated the effects of NGF on stroke. The transplantation of bone mesenchymal stem cells (BMSCs) with NGF via the tail vein in MCAO rats was found to improve neurological function and promote the differentiation of BMSCs [124]. Moreover, the intranasal administration of NGF in rat MCAO model improved neurological function with a significant reduction in the infarct volume and enhancement in survival and proliferation of progenitor cells [128].

### Design considerations for tissue engineering approach

The engineered scaffolds can be rationally designed to fulfill different regeneration requirements for different tissues or organs. For effectively treating stroke, some general criteria need to be met when one chooses a biomaterial. First of all, an ideal scaffold biomaterial for stroke tissue regeneration should bear a three-dimensional structure that provides a highly biocompatible microenvironment favoring cell growth, adhesion, migration, proliferation and differentiation without eliciting inflammatory responses *in vivo*[17]. Second, the sufficient number of appropriate cells are required to initiate regeneration and repopulate the affected neuronal tissue towards function restoration. Third, a key aspect for functional replacement of damaged brain tissue is to control the differentiation of transplanted cells into desired phenotypes and guide their integration with the host parenchyma to replace and replenish the damaged neuronal population. This can be achieved through the use of appropriate growth factors. Thus, the biomaterial is required to be able to carry and release these factors *in vivo*, which should be realized in a controlled manner as the controlled release has been proven to enhance therapeutic effects.

More importantly, by modifying physical and chemical traits of the biomaterial, the engineered scaffold can be designed to possess diverse unique properties. For example, the dynamics of scaffold biodegradation can be programmed to synchronize with the host healing process [129]. Degradable scaffolds can serve as a temporary delivering vehicle for cells and growth factors while avoiding the chronic problems caused by long-term biomaterial implantation. Scaffolds can be designed to acquire the shape-memory property [129] that allows the scaffolds to be transplanted through a minimally invasive approach. Fine modulations of chemical composition of a scaffold can achieve controlled release of the drugs encapsulated within the scaffold *in vivo*, which would enhance the therapeutic effects of these drugs. When such scaffolds are integrated with cell and cytokine therapies, their unique properties would help to overcome the inherent limitations of these therapies. Additionally, when one designs a scaffold-based tissue engineering strategy, the host immune response to the scaffold, host tissue microenvironment, such as local angiogenesis and vascularization, should be also taken into consideration. Combining these design considerations into scaffold fabrication would maximize the advantages of the tissue engineering approach for stroke treatment.

## Conclusions

The need to develop effective therapeutic approaches for the treatment of stroke is compelling. However, to structurally and functionally restore the damage caused by ischemic stroke remains challenging in part because the brain is the most complex organ [44].

Owing to its powerful potential in facilitating tissue regeneration, the tissue engineering based strategy is becoming another promising approach for ischemic stroke treatment. A number of different types of stem cells, including embryonic stem (ES) cells, iPS cells and NSCs, have been utilized in cell therapy to repair the injured brain tissue [90]. One of the major problems associated with this approach is poor cell survival. We believe that with the aid of appropriate scaffolds as carriers for cells, cell survival will be greatly improved *in vitro* and *in vivo*.

Some biomaterial scaffolds have been examined in stroke regeneration, such as collagen, hyaluronan, matrige, laminin and nanomaterials (Table 2) [16]. The results indicate biomaterial scaffolds hold promise for promoting structural and functional restoration of stroke-damaged neuronal tissue.

Growths factors have been applied in stroke regeneration such as BDNF, GDNF and EPO [73, 95, 97]. These growth factors can reduce the volume of infarct areas and induce stem cell differentiation (Table 3). However, the intravenous administration often results in a therapeutic effect that is rather transient and inefficient, because some growth factors cannot effectively pass through the blood brain barrier. Localized and sustained release of growths factors or therapeutic factors via scaffolds can be a solution to this problem.

Meanwhile, alternative sources of cells and new combinations of neuro-protective factors should be explored. Advances in development of new scaffold materials may bring tissue engineering treatment one step closer to clinical applications.

Taken together, the tissue-engineering strategy complements cell and cytokine therapies. The combination of both can be a valuable alternative for the clinical treatment of ischemic stroke.

## Abbreviations

tPA: tissue-type plasminogen activator
NSCs: Neural stem cells
CNS: Central nervous system
SVZ: Subventricular zone
MCAO: Middle cerebral artery occlusion
hNSCs: human neural stem cells
MSCs: mesenchymal stem cells
FGF2: Fibroblast growth factor-2
EGF: Epidermal growth factor
BDNF: Brain-derived neurotrophic factor
GFAP: Glial fibrillary acidic protein
SGZ: Subgranular zones
iPS: induced pluripotent stem
HAMC: Hyaluronan/methyl cellulose
EPO: Erythropoietin
NGF: Nerve growth factor
PRAS: Proline-rich Akt substrate
YWHAQ: tyrosine 3-monooxygenase/tryptophan 5-monooxygenase activation protein, theta polypeptide
pAkt: phosphory-lated Akt
ECM: Extracellular matrix
PLGA: Poly (D, L-lactic acid-co-glycolic acid)
ECs: Endothelial cells
NPCs: Neural progenitor cells
GDNF: Glial cell line-derived neurotrophic factor
rhEPO: recombinant human erythropoietin
ES: Embryonic stem
TGF-β: Transforming growth factor-β
TrkB: Tropomyosin receptor kinase B.
